# Ascorbate Preferentially Stimulates Gallium-67 Uptake in Glioblastoma Cells

**Published:** 2022-07-18

**Authors:** Michael S. Petronek, M. Li, J.N. Sarkaria, M.K. Schultz, B.G. Allen

**Affiliations:** 1 Department of Radiation Oncology, University of Iowa; Iowa City, IA, USA; 2 Viewpoint Molecular Targeting, Inc., Coralville, IA USA; 3 Department of Radiation Oncology, Mayo Clinic; Rochester, MN, USA; 4 Department of Radiology, University of Iowa; Iowa City, IA, USA

**Keywords:** Gallium-67, *In vitro*, Cells

## Abstract

Gallium is a tri-valent p-block metal that closely mimics tri-valent iron. Gallium is internalized into cells *via* transferrin receptor-mediated endocytosis. Both Ga-67 and Ga-68 are radionuclides that can be radiolabeled to various bioactive compounds for clinical imaging procedures to visualize tumors and sites of inflammation. High-dose ascorbate (pharmacological ascorbate) is an emergent glioblastoma therapy that enhances cancer cell-killing through iron-metabolic perturbations. We hypothesized that pharmacological ascorbate treatments might alter Ga-67 uptake in glioblastoma cells. We evaluated the *in vitro* ability of pharmacological ascorbate to alter gallium uptake in patient-derived glioblastoma cells with variable genetic backgrounds by co-incubating cells with Ga-67 ± pharmacological ascorbate. Surprisingly, we observed increased basal gallium uptake in the glioblastoma cells compared to normal human astrocytes. Further, pharmacological ascorbate treatment stimulated gallium uptake in glioblastoma cells while not affecting uptake in normal human astrocytes. This effect appears to be related to transient increases in transferrin receptor expression. Finally, pharmacological ascorbate treatment appears to stimulate gallium uptake in an iron metabolism-dependent manner. Further mechanistic experiments are required to evaluate the translational utility of ascorbate to impact gallium tumor imaging.

## Introduction

Gallium (Ga) is a group 13 metal that has similar chemical properties and behavior to trivalent iron (Fe). The major difference between Ga and Fe is in its valence d-orbital shell. Ga^3+^ is a d^10^ metal that has 10 paired d-orbital electrons with a resulting net spin of 0 (S = 0) while Fe^3+^ is a d^5^ metal with 5 unpaired d-orbital electrons in a high-spin state (S = 5/2). This difference in electron composition allows Fe^3+^ to transfer electrons and undergo redox cycling while Ga^3+^ is largely redox inactive [1]. However, Ga^3+^ and Fe^3+^ can be coordinated similarly [2]. For example, Ga^3+^ can mimic Fe^3+^ and bind to transferrin receptors to be internalized by cells *via* transferrin receptor (TfR)-mediated endocytosis [3,4]. TfR-mediated endocytosis is thought to be the primary uptake mechanism for Ga-based imaging studies [5].

Ga is routinely used in radiolabeling bioactive molecules for clinical imaging. The two most common isotopes of Ga used in imaging are Ga-67 and Ga-68. Ga-67 is a γ-emitter with a half-life of 3.26 d while Ga-68 is a positron emitter (≈89% yield; t_1/2_ = 68 min.) [6]. Both isotopes of Ga are useful for tumor imaging studies but Ga-67 may also be used to evaluate sites of inflammation [5,7,8]. Ga is believed to be preferentially taken into tumor cells relative to their surrounding normal tissues due to the increased Fe demands of tumor cells [9]. In 1972, Chen DCP, et al. observed that Ga-67 was preferentially internalized in tumor cells relative to normal cells; Ga internalization was improved by transferrin supplementation in the cell culture media [10]. An analysis of 17 WHO grade III or IV glioma patients imaged with ^68^Ga-citrate demonstrated that Ga preferentially localizes within the tumor and corresponds with the MR-detectable lesions in patients with suspected pseudoprogression [5]. This study also showed that ^68^Ga-citrate is internalized by U87 glioblastoma (GBM) subcutaneous tumors in a TfR expression-dependent manner [5]. Thus, Ga-based imaging may be a tool to evaluate for Fe metabolic changes in high grade gliomas. However, the effect of high doses of ascorbate (pharmacological ascorbate; gram intravenous infusions of ascorbate resulting in millimolar plasma ascorbate concentrations) on the mechanisms that drive Ga internalization has yet to be investigated.

Pharmacological ascorbate has recently emerged as a novel, iron-dependent therapeutic strategy in GBM. In a phase I trial of 11 patients receiving 87.5 g infusions three times weekly with concurrent temozolomide and ionizing radiation, median progression-free survival and overall survival was 9.4 and 18 months, respectively [11]. Historically, GBM patients treated with standard of care ionzing radiation and temozolomide have progression-free survivals and overall survivals of 6 and 14.6 months respectively [12,13]. The effective enhancement of standard of care therapies by pharmacological ascorbate has also been observed in pre-clinical models of GBM [14]. The observed enhancement of radiation and temozolomide by pharmacological ascorbate is largely attributed to its ability to generate large fluxes of H_2_O_2_ leading to subsequent perturbations of iron metabolism and increased labile iron capable of catalyzing oxidation reactions and enhancing cell death [14,15]. Because TfR expression is a critical component of the global Fe metabolic network, we hypothesize that pharmacological ascorbate will alter Ga uptake in GBM cells.

## Materials and Methods

### Cell culture and Ga-67 measurement

Patient-derived GBM cell lines (GBM06, GBM14, and GBM39) were kindly provided by Dr. Jann Sarkaria (Mayo Clinic; Rochester, MN). GBM06 and GBM39 cell lines were derived from a primary tumor while GBM14 was derived from a recurrent tumor. Their cellular characteristics can be found in (Table 1). GBM cell lines and normal human astrocytes (NHAs) were cultured at 21% O_2_ and grown to 70–80% confluence prior to experimentation. Cells were cultured in DMEM-F12 media (15% FBS, 1% penicillin-strep, 1% Napyruvate, 1.5% HEPES, 0.1% insulin, and 0.02% fibroblast growth factor). Cells were treated with 200 μCi GaCl_3_ ± 10 pmol cell^−1^ ascorbate at 21% O_2_ for up to 3 h. Ga-citrate was generated by diluting GaCl_3_ at a 1:1 ratio with citric acid in phosphate-buffered saline. Cells were washed with 1X PBS and lysed before gamma-counter analysis to ensure that only the internalized Ga-67 was being counted.

### Western blotting

Cells were harvested in 1X lysis buffer containing a phosphatase inhibitor cocktail. Total protein (25 μg) was electrophoresed on a 4–20% gradient gel (Bio-Rad) at 150 V for approximately 1.5 hr. The separated proteins were transferred onto PVDF membranes (Millipore, Billerica, CA) and non-specific binding was blocked using 5% nonfat dry milk in PBS-Tween (0.2%) for 1 hr at room temperature. The membranes were incubated with Transferrin receptor primary antibody (1:1000, Invitrogen, Camarillo, CA) at 4°C overnight. b-actin served as a loading control (1:4,000; Sigma-Aldrich). Following three 5 min PBS-Tween washes, the membranes were probed with secondary antibodies (mouse anti-rabbit; 1:25,000; Sigma-Aldrich, St. Louis, MO) that were conjugated with horseradish peroxidase for 1 hour. The washed membranes were incubated with Super Signal West Pico Chemiluminescent Substrate (Thermo Scientific, Rockford, IL) and exposed to CareStream BioMax MR Film (CareStream Health, Rochester, NY).

### Statistical considerations

All experiments were performed in triplicate. Statistical analysis was performed using Graphpad prism software where pairwise comparisons were performed using a Welch’s T-test assuming unequal variances in each group. For experiments with multiple groups, one or two-way ANOVA tests were performed, and multiple comparisons were done with a Tukey’s post-hoc analysis.

## Results

### Baseline Ga-67 uptake

**Figure 1.**
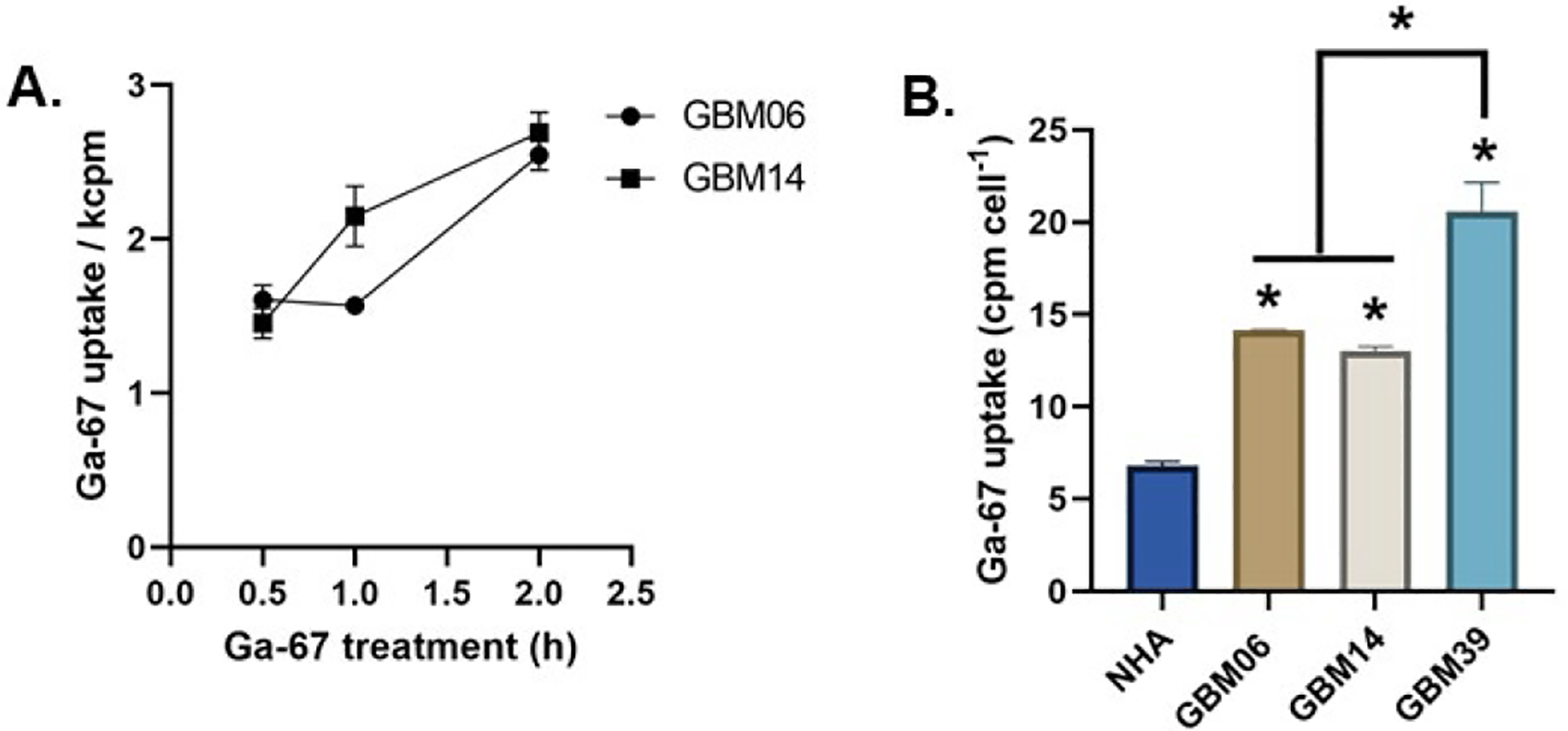
Baseline Ga-67 uptake in GBM and normal cells. **A.** GBM06 and GBM14 cells were incubated with 200 μCi GaCl_3_ and harvested at multiple time points to evaluate temportal Ga uptake. **B.** NHA, GBM06, GBM14, and GBM39 cell lines were incubated with 200 μCi GaCl_3_ for 3 h prior to cell harvesting and analysis. Error bars represent SD of three experiments. One-way ANOVA with Tukey’s post-hoc analysis was performed with *p<0.05.

To optimize our approach for studying Ga uptake, we evaluated the temporal dependence of Ga-67 uptake. We utilized Ga-67 in this study primarily due to its long half-life (t_1/2_ = 78.3 h) allowing for efficient utilization in *in vitro* studies. We were able to determine that in both GBM06 and GBM14 cell lines; there is continuous uptake of Ga-67 over a 2 h period (Figure 1A).

Due to the recent reports of preferential accumulation of Ga in glioma tumors, we aimed to validate this in our study (5). Following a 3 h incubation with Ga-67, we found the three different patient-derived cell lines (GBM06, GBM14, and GBM39) have significantly higher Ga-67 uptake relative to normal human astrocytes (NHA; Figure 1B). GBM06 and GBM14 cell lines exhibit ≈ 2.5-fold higher Ga-67 uptake while GBM39 has significantly greater uptake than either GBM06 or GBM14 (≈ 4-fold). Interestingly, the major difference between the cell lines is that GBM39 is a mesenchymal subtype while GBM06 and GBM14 are classical (Table 1). This suggests that mesenchymal GBM cells may have a greater Fe dependence as compared to their classical counterparts, consistent with previously reported literature on mesenchymal GBM cells being more aggressive and having stem cell-like phenotypes that are reported to have increased Fe uptake [16,17].

### Effects of pharmacological ascorbate on Ga-67 uptake

**Figure 2.**
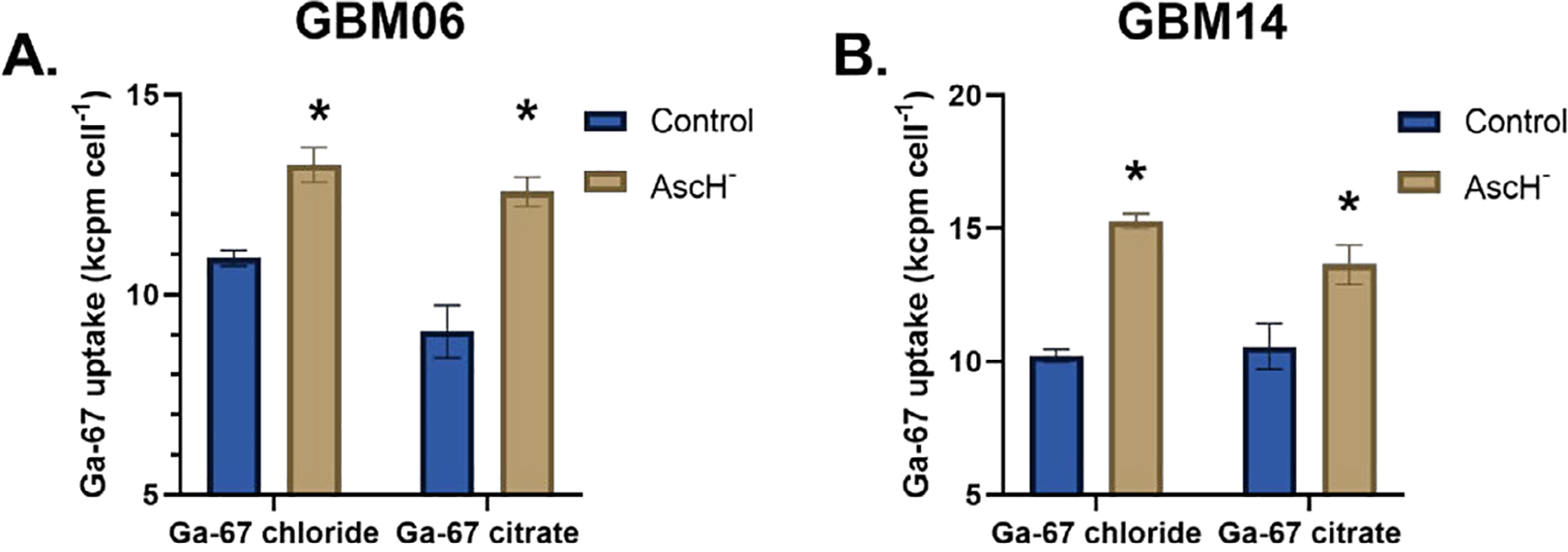
Ascorbate stimulates Ga-67 uptake in patient-derived GBM cells. GBM06 (**A**) and GBM14 (**B**) cells were incubated with 200 μCi GaCl_3_ or Ga-citrate ± 10 pmol cell^−1^ Pharmacological ascorbate (AscH^−^) for 3 h prior to harvesting and analysis. Individual groups were compared using a Welch’s T-test with *p<0.05.

**Figure 3.**
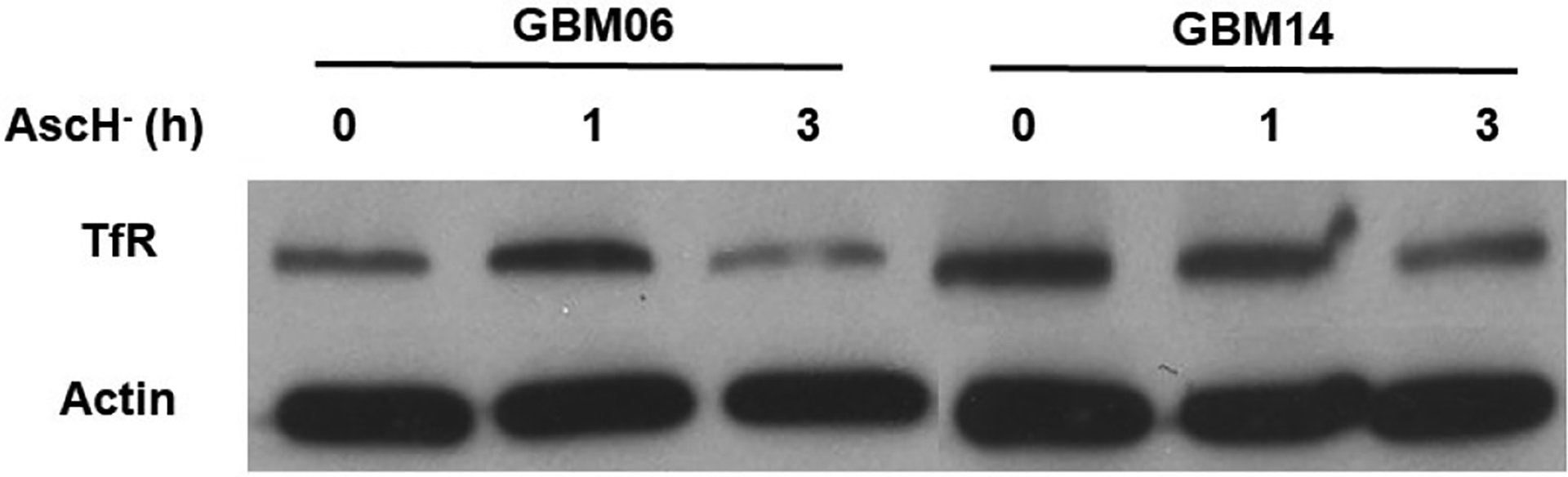
Pharmacological ascorbate alters TfR expression in patient-derived GBM cells. GBM06 and GBM14 cells were incubated with 10 pmol cell^−1^ Pharmacological ascorbate (AscH^−^) and harvested to evaluate TfR expression temporally. Membranes were incubated with Transferrin receptor primary antibody (1:1000, Invitrogen, Camarillo, CA) at 4°C overnight. b-actin served as a loading control (1:4,000; Sigma-Aldrich).

Both GBM06 and GBM14 cells were co-incubated with Ga-67 ± pharmacological ascorbate (10 pmol cell^−1^) for 3 h to allow for continuous Ga-67 uptake. In both cell lines, treatment with pharmacological ascorbate stimulated a significant increase in Ga-67 uptake (Figures 2A and B). The Ga-67 treatment was done with both GaCl_3_ and Ga-citrate to determine if this effect was ligand-specific; Ga-67 is commonly bound to citrate for *in vivo* studies and Ga-67 citrate is regularly utilized for both tumor and inflammation imaging [18,19]. Both GaCl_3_ and Ga-citrate showed increased Ga-67 uptake. Ga-citrate imaging is also an accepted approach for Fe-mimic studies in cell culture [20]. In our study, pharmacological ascorbate stimulated both GaCl_3_ and Ga-citrate uptake. Thus, this effect appears to be ligand-independent. The primary mechanism of Ga uptake is believed to be *via* TfR-mediated endocytosis [3]. To evaluate the effects of pharmacological ascorbate on TfR stability in both GBM06 and GBM14 cells, we assessed TfR stabilization by western blotting. We observed increased TfR stabilization in GBM06 cells following a 1 h ascorbate treatment; however, a 3 h ascorbate treatment resulted in TfR instability (Figure 3). Similar results were obtained with GBM14 cells. These results suggest that the increased Ga-67 uptake is likely the result of transient TfR stabilization by pharmacological ascorbate; however, these results are correlational and further mechanistic studies are required to establish a causal relationship.

**Figure 4.**
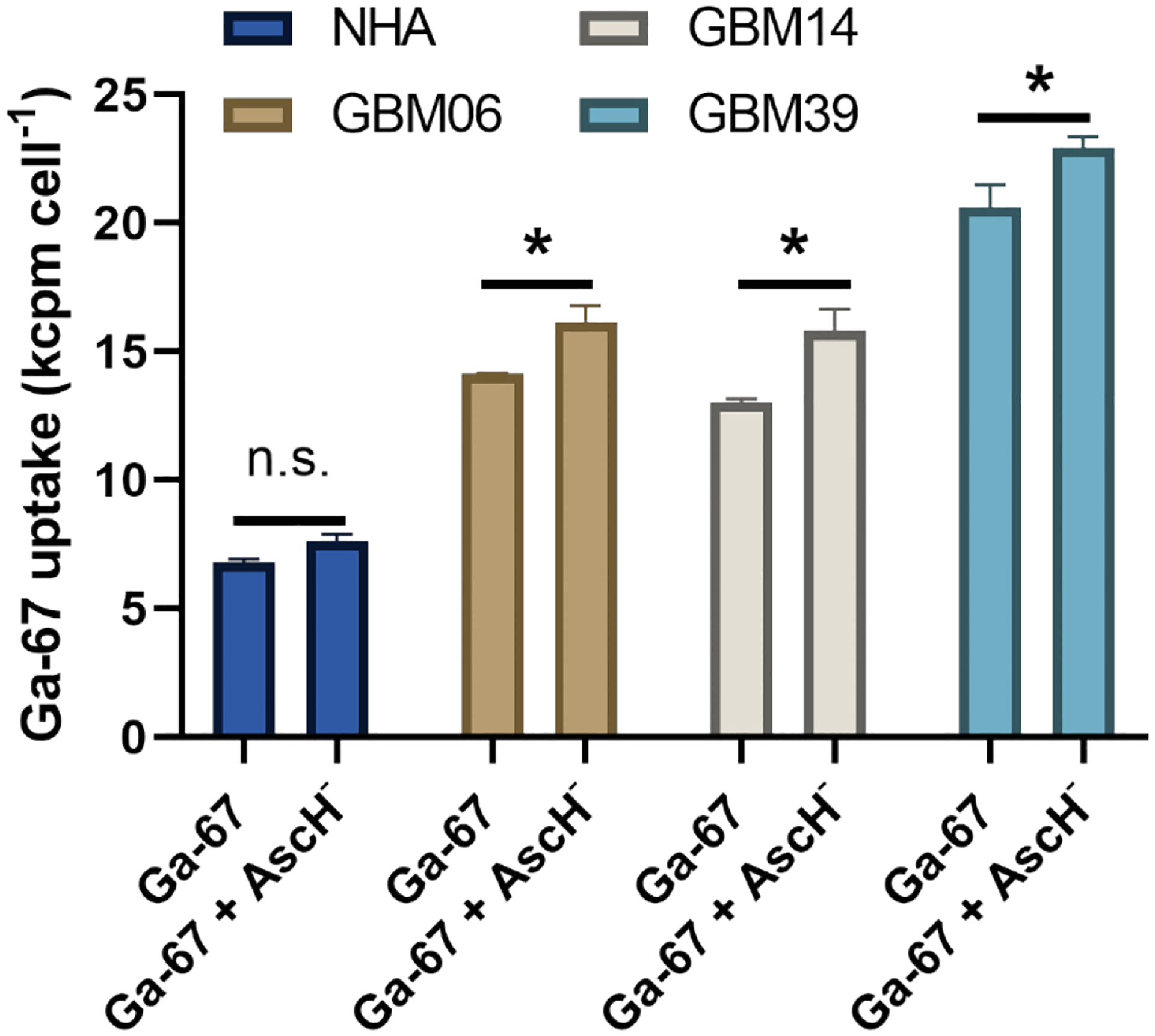
Stimulated gallium uptake by ascorbate is specific to GBM cells. NHA, GBM06, GBM14, and GBM39 cells were incubated with 200 μCi GaCl_3_ ± 10 pmol cell-1 Pharmacological ascorbate (AscH^−^) for 3 h prior to harvesting and analysis. Error bars represent SD of three experiments. Two-way ANOVA with Tukey’s post-hoc analysis was performed with *p<0.05.

Lastly, we aimed to determine if pharmacological ascorbate enhanced Ga-67 uptake is specific to GBM cells relative to normal human astrocytes. We incubated the three patient-derived GBM cell lines (GBM06, GBM14, and GBM39) and normal human astrocytes for 3 h with Ga-67 ± pharmacological ascorbate. All three GBM cell lines showed a significant increase in Ga-67 uptake while normal human astrocytes showed no significant change in uptake (Figure 4). Thus, the stimulation of Ga-67 uptake by pharmacological ascorbate appears to be specific to GBM cells relative to their normal cell counterpart.

## Discussion

In this study, we report the novel, preliminary observation that pharmacological ascorbate can preferentially stimulate Ga uptake in GBM cells. These observations are important because it suggests the potential for improved gallium imaging of GBM tumors when combined with pharmacological ascorbate ascorbate. Pharmacological ascorbate and Ga may also serve as a novel theranostic pairing well-suited for further investigation in GBM.

From a basic, Fe metabolic perspective, these data suggest that pharmacological ascorbate may stimulate transferrin-bound Fe uptake. It is well established that Ga binds to transferrin and is internalized *via* TfR-mediated endocytosis [2,3]. Ga has been shown to be preferentially internalized into human GBM tumors cells and uptake is directly related to the tumor TfR expression [5]. Beyond that, Ga-maltolate can mimic Fe and disrupt Fe metabolism in GBM tumor cells [21].

**Figure 5.**
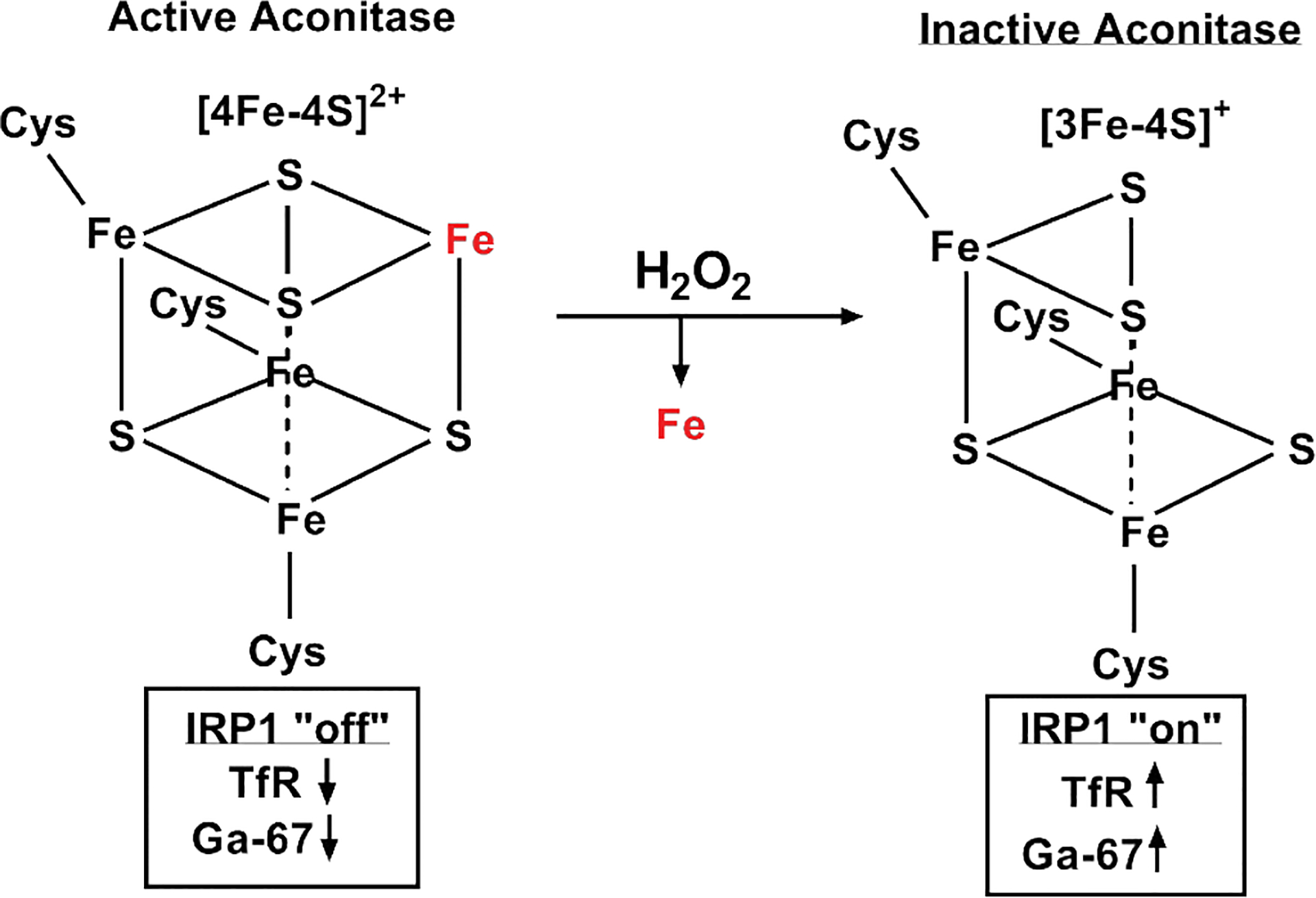
Hypothetical, iron metabolic model for ascorbate-stimulated Ga-67 uptake. The cytotoxic effects of ascorbate generally occur in an H_2_O_2_ - dependent manner. Therefore, we hypothesize that ascorbate can stimulate Ga-67 uptake by altering iron responsive protein activities. More specifically, H_2_O_2_ can remove the solvent exposed Fe^2+^ of aconitase in a Fenton chemistry - like manner. In doing so, it leaves an incomplete [3Fe-4S]^+^ cluster that may be able to function as iron responsive protein 1 (IRP1) leading to enhanced transferrin receptor (TfR) mRNA stabilization and Ga-67 uptake.

Similarly, pharmacological ascorbate can alter Fe metabolism by increasing redox active labile Fe levels [14,22]. What is less clear is the impact pharmacological ascorbate has on critical Fe metabolic features, such as ferritin or TfR, that are necessary in maintaining Fe homeostasis. What is known is that pharmacological ascorbate induced H_2_O_2_ fluxes decrease aconitase activity in GBM cells [14,23]. Aconitase is a critical iron-sulfur cluster enzyme that functions in the citric acid cycle and its cytosolic form serves as iron-responsive protein 1 (IRP1) (24). The [4Fe-4S]^2+^ cluster of aconitase is sensitive to oxidation by H_2_O_2_, which leads to an incomplete [3Fe-4S]^+^ cluster and enzymatic inactivation [24–26]. When aconitase functions as IRP1, it can bind to an iron response element on the 3’ end of TfR mRNA leading to stabilization and enhanced expression [27]. Therefore, these results present a novel, testable hypothesis that pharmacological ascorbate induced H_2_O_2_ fluxes oxidize the [4Fe-4S]^2+^ cluster of aconitase leading to IRP1 activation, enhanced TfR mRNA stabilization, and increased transferrin-bound Fe (Ga) uptake (Figure 5). Further mechanistic studies are required to investigate this postulate. The GBM cell specificity we observed is consistent with previous reports that the large H_2_O_2_ fluxes generated by pharmacological ascorbate treatment are more readily able to be handled by normal cells that have increased levels of catalase and peroxidases (e.g. glutathione peroxidase 1) [14,23].

From a clinical imaging perspective, the tumor-specific enhancement of Ga uptake by pharmacological ascorbate may provide enhanced image contrast between GBM tumors and surrounding normal brain tissue. Inducing changes in Ga internalization may also reveal new insights on the biological status of GBM tumors. When using Ga-based MRI to assess treatment progression, the inability to differentiate between true tumor progression and damaged normal tissue due to the radiation (i.e. pseudoprogression) can frequently lead to unnecessary surgical resections [28,29]. At baseline, we have observed *in vitro* that Ga-67 uptake is significantly greater in GBM cells as compared to NHA. This finding is consistent with human studies that investigated Ga-based imaging as a method of differentiating GBM and normal brain tissue [5]. Because pharmacological ascorbate stimulates Ga uptake preferentially in GBM cells relative to normal human astrocytes, pharmacological ascorbate may be a rapidly translatable approach to enhance PET (Ga-68) image contrast in GBM patients. Thus, the utilization of a single pharmacological ascorbate infusion prior to a Ga-68 PET scan could provide a significant advancement in the diagnosis and management of pseudoprogression in GBM patients.

## Conclusion

In summary, we have observed in patient-derived GBM cells that pharmacological ascorbate is able to stimulate Ga-67 uptake. This effect appears to be specific to GBM cells as normal human astrocytes remained largely unaffected. Ga-67 uptake corresponds with transient TfR stabilization in GBM cells which we hypothesize may be the result of H_2_O_2_ – mediated alterations in IRP1 activity. These *in vitro* results provide novel preliminary insights that warrant further mechanistic studies into the effects of pharmacological ascorbate on Ga tumor imaging.

## Figures and Tables

**Table 1.** Molecular characteristics of patient-derived GBM cell lines used in this study.

Cell line	Primary or Recurrent	Molecular Subtype	Molecular Subtype	EGFR Amplification (Mutation)	MGMT Status	IDH1/2 Status
GBM06	Primary	Classical	Classical	Yes (VIII)	Methylated	Wildtype
GBM14	Recurrent	Classical	Classical	No	Unmethylated	Wildtype
GBM39	Primary	Mesenchymal	Mesenchymal	Yes (VIII)	Methylated	Wildtype
